# The Effects of Ethnic Identity on Discrimination and Depression and Anxiety in a Sample of Arab American Adults

**DOI:** 10.1002/jcop.70068

**Published:** 2025-12-02

**Authors:** Molly Green, Ken Resnicow, Elizabeth J. King, Madiha Tariq, Asraa Alhawli, Minal Patel

**Affiliations:** ^1^ Department of Health Behavior & Health Education University of Michigan School of Public Health Ann Arbor Michigan USA; ^2^ Present address: Elon University‐Public Health Studies Ann Arbor Michigan USA; ^3^ University of Michigan Rogel Cancer Center Elon North Carolina USA; ^4^ Present address: University of Minnesota‐Division of Epidemiology & Community Health Minneapolis Minnesota USA; ^5^ Arab Community Center for Economic and Social Services Dearborn Michigan USA; ^6^ Present address: Oakland County Executive Office Building 41W Waterford Michigan USA

**Keywords:** Arab Americans, depression and anxiety, discrimination, ethnic identity, identity, mental health

## Abstract

The aim of the study was to clarify how ethnic identity may impact poor mental health outcomes related to discrimination among Arab American adults in Southeast Michigan, USA. 286 respondents completed a health attitudes and behaviors survey. We used structural equation modeling with path and multi‐group analyses to examine moderation effects of ethnic identity on the relationship between discrimination and depression and anxiety, and further moderation based on gender. Ethnic identity positively buffered against depression and anxiety associated with discrimination. In the subgroup analysis, ethnic identity was protective for female participants, though not male participants. These findings provide evidence for ethnic identity as a buffer between discrimination and poor mental health among Arab American adults. Mechanisms may include feelings of belonging and social support. The stronger effects for women may be due to their role of transmitting cultural and religious traditions. Future interventions should incorporate ethnic identity as a protective feature for mental health.

## 1 Introduction

Arab Americans are a small but growing population in the US estimated between 1 and 3.7 million (Arab American Institute Foundation 2021) and are an important ethnic minority population in Michigan. Until very recently Arab and other Middle East and North African (MENA) Americans were not officially recognized as a racial/ethnic minority by the US government and have been considered “White” in the US census and other surveys of population and health. The addition of a MENA category to the Office of Management and Budget racial/ethnic minority categories will take effect for the 2030 census (Marks et al. 2024). Until this change comes into effect, our understanding of the health and well‐being of this community is limited (Awad et al. 2022; Resnicow et al. 2022). The existing data, mainly from small studies in Michigan where there is an Arab ethnic enclave, indicate that the privileges of whiteness has not been extended to many Arab Americans and health inequities abound (Abuelezam et al. 2018). Arab Americans in Michigan and nationally experience high levels of discrimination and other identity‐based stressors, comparable to other ethnic minority groups (Awad et al. 2019; Ikizler and Szymanski 2018). Compared to majority white Americans in Michigan, Arab Americans have shown significantly worse mental health outcomes, including depression (Samari et al. 2018). Discrimination has been linked to poor mental health in this population, including depression and low levels of psychological well‐being (Abuelezam et al. 2018).

### 1.1 Ethnic Enclaves and Mental Health

Ethnic enclaves are present throughout the US, including the Cuban enclave in Miami, Chinese enclaves throughout California and major US cities (Waters and Eschbach 1995). For immigrants and their families, living in ethnic enclaves can help mitigate the negative effects of the acculturation process (Kang et al. 2009), and can provide an alternative space when discrimination and other negative elements are present (Portes and Zhou 1994). Researchers have found that among immigrants, strong feelings of community and social support are protective for health. These reinforce worldviews, traditions, and values and create a sense of connectedness and belonging, which in turn can promote a sense of value and good mental health (Yoon et al. 2013).

Residence in ethnic enclaves can also be negative for health. For some residents of ethnic communities, pressures to maintain their ethnic culture have been associated with negative mental health outcomes (Kim et al. 2014). Communities with significant concentrations of ethnic minorities often also contain substandard housing, pollution exposure, inadequate health services, and other indicators of low socioeconomic conditions (Patel et al. 2003).

For Arab Americans in SE Michigan, a history of migration to the area and strong community ties; economic networks; family connections; and possible ease of transition, including language and cultural familiarity, have led to the creation and maintenance of an Arab ethnic enclave (Abraham et al. 2011). There are important demographic differences in the ethnic enclave compared to nearby areas and the rest of Michigan. In Dearborn, the most recently reported high school graduation rate is between 90% and 97%, compared to 81.4% statewide (Leeds 2021). While the median income in Dearborn, $49.8k–$84 K, is similar to nearby cities, the percentage of children living in poverty is higher in Dearborn than in neighboring cities at 36.2%–47.6% (Center for Urban Studies at Wayne State University 2021). In the city, over 29% of the population is under 18 years of age, higher than the national average of 22.3%. Nearly 30% of the population in Dearborn identified as foreign‐born, compared to 6.9% in Michigan as a whole (United States Census Bureau 2021). Some of these demographic characteristics that differentiate Dearborn from the rest of the state help to create a strong sense of community and identity for its residents. Community values, such as education and family, life are reflected in these statistics and can add to the sense of belonging and shared culture (Jang et al. 2015). However, others, like the percentage of children living in poverty, demonstrate that economic success can be difficult to achieve, especially for communities with large percentages of immigrants, due to limited English‐language skills and the abundance of low‐skilled, low‐paying jobs, among other factors (Gold 2015).

### 1.2 Discrimination, Ethnic Identity, and Mental Health

Discrimination has been well‐established as a substantial, chronic stressor for other ethnic minority groups in the US, including African Americans, Latinos, and Asian Americans (Carter et al. 2017). For many Arab Americans, their ethnic identity and religion play a prominent role in their experiences of discrimination (Awad 2010). Though Muslims are a minority of the Arab American community in the US (Awad et al. 2019), they may be more likely to be subjects of discrimination, especially because of visible religious identifiers, including hijabs and beards, (Ikizler and Szymanski 2018), the racilization of Muslim identity, and associations with 9/11 and the War on Terror (Awad et al. 2022). Discrimination may also be more prevalent for sub‐groups within the Arab American enclave community, including younger individuals (Kader et al. 2019) who may better discern discriminatory elements in the social environment (Green et al. 2024), and those and those with intersectional identites such as those who identify as Black and Muslim who may experience discrimination based on both religious and racial identities (Kaufman and Niner 2019). A facet of discrimination which is somewhat unique in this community are the simultaneous conditions wherein Arab Americans experience substantial discrimination but are also invisible in health and population data due largely to their unrecognized status as an ethnic minority in the US (Awad et al. 2022). This lack of data presents a considerable barrier for understanding discrimination and mental health outcomes. Compounding these issues, Arab Americans in SE Michigan may experience significant stigma in screening and treatment for mental health issues (Dallo et al. 2018).

Among protective factors, researchers have found that a strong ethnic identity is an important promoter of well‐being for ethnic minorities (Atari and Han 2018), including Asian Americans and Latinos (Espinosa et al. 2018). Ethnic identity has been theorized to encompass multiple elements including a sense of belonging to one's ethnic group and self‐identifying as a part of it; commitment to this group; and cultural behaviors, beliefs, and values. Ethnic identity is generally conceptualized as complex and multi‐faceted, and an individual's sense of ethnic identity can develop and change over time (Phinney and Ong 2007). The findings from the limited work that has been done around the connections between ethnic identity and well‐being for Arab American youth and young adults in SE Michigan (Kumar et al. 2015; Seff et al. 2021) and adults outside the ethnic community (Atari and Han 2018; Alsaidi et al. 2023) suggest that ethnic identity may also be protective of and promotive for well‐being for Arab Americans, as it can be for other ethnic minority populations. Coping with discrimination may include utilizing methods that incorporate ethnic identity and an individual's ethnic community.

A strong sense of ethnic identity can also protect against the stress of discrimination through the utilization of support‐seeking coping mechanisms (Phinney and Chavira 1995) or as protection against maladaptive coping behaviors (Chae et al. 2008). Previous research shows the protective effects of ethnic identity are, however, mixed. Some evidence suggests a potential exacerbating rather than buffering role of ethnic identity on the relationship between discrimination and poor mental health outcomes (Thibeault et al. 2018). This may be a result of strong identification with a group, paired with negative feelings about and associations towards that same group. Discrimination could then reinforce those negative feelings and self‐regard, leading to poor mental health (Phinney 1991; Atkin and Tran 2020; Crichlow et al. 2024). A strong sense of ethnic identity may also sensitize an individual to perceiving discrimination, whereas those with a weaker sense of ethnic identity may be less prone to perceiving discrimination. Individuals may perceive discrimination to be more racially based or may be more impacted by discrimination that affects an aspect of their identity that they see as more central (Bombay et al. 2010). The role of ethnic identity as a potential moderator of the relationship between discrimination and mental health has not previously been explored among Arab American adults within the Arab ethnic community in SE Michigan.

### 1.3 The Role of Gender in Ethnic Identity, Discrimination, and Mental Health

Expectations and experiences of gender within the Arab American community differ from the dominant US culture. In this community, and especially within the ethnic enclave in Dearborn, women play a unique role in holding and transmitting the Arab culture and their religious traditions to future generations (Ajrouch 2004; Read and Oselin 2008). Indeed, female participants in one study in the Detroit metro area reported a stronger commitment to both religious and ethnic practices than their male counterparts (Samari 2016), and higher private regard of their ethnic identity than men in another recent national study (Ahmed et al. 2022). Experiences of discrimination can also differ for men and women in the Arab American community. Muslim Arab American women may have an elevated risk of experiences discrimination, especially because they are often visibly identifiable as Muslim due to the common practice of wearing the hijab (Gulamhussein and Eaton 2015), though discrimination can be of an intersecting nature targeting gender, racial, and religious identities (Alsaidi et al. 2023). In one study in the Chicago area, Arab American women reported experiencing more than two times as many hate crimes as men, and in 90% of these crimes, a woman wearing a hijab was present (Cainkar 2009). Importantly, researchers have also detected differences in mental health within the Arab American community in Michigan, notably in findings of significantly higher levels of depression for women compared to men (Samari et al. 2018). While gender roles and expectations, discrimination, and mental health can differ for men and women in the Arab American community, it is unclear whether a sense of ethnic identity differs for women and men or has differential influences on mental health outcomes.

### 1.4 Hypotheses

Insufficient data on mental health outcomes for Arab American adults have resulted in an incomplete understanding of the mechanisms through which experiences of discrimination may result in symptoms of depression and anxiety and whether a sense of ethnic identity impacts this relationship, as well as how Arab American adults may understand and cope with discrimination and promote or protect good mental health. Apart from Seff and colleague's (2021) study with adolescents, studies of discrimination and poor mental health outcomes among Arab Americans do not provide further insight into the role of residence in an ethnic enclave in these mental health outcomes and none of them address the possible role of ethnic identity within the enclave community. While Arab Americans may be at risk for poor mental health outcomes, there may also be protective and promotive factors for mental health in the Arab American community in SE Michigan, including ethnic identity affirmation (Atari and Han 2018; Ikizler and Szymanski 2018). There is a lack of understanding of how these possible protective and promotive elements may act as mechanisms for good mental health in the Arab American community, particularly in a socio‐political climate where Arab Americans are villainized. Further understanding of these pathways can provide evidence to be used in the development and planning of interventions and other programming focused on mental health in the Arab American community, a much‐needed next step for improving mental health.

To the best of our knowledge, no studies have yet examined the potential buffering effects of ethnic identity on the relationship between discrimination and mental health outcomes among Arab American adults with the ethnic enclave community in SE Michigan. Our objective was to examine how ethnic identity may impact the link between discrimination and mental health among Arab American adults. We tested the hypothesis that ethnic identity (EI) would alter the relationship between perceived discrimination and latent depression and anxiety, such that it is protective against poor mental health. We further examined if these effects would differ by gender between men and women. Initially, we also hypothesized that immigration status may also play a role in these relationships, but the model showed no main or interaction effects, possibly due to the overarching effects of ethnic identity. In terms of main effects, we hypothesized that there would be a positive association between discrimination and poor mental health outcomes.

## 2 Materials and Methods

### 2.1 Study Design, Data Sources, and Study Population

Data are from a cross‐sectional convenience sample survey exploring health related knowledge, attitudes, and behavior of MENA adults living in SE Michigan. The survey items were translated into Arabic and reviewed by professionals who are bilingual in English and Arabic at the Arab Community Center for Economic and Social Services (ACCESS). Between May and September 2019, a survey, available in English and Arabic, was distributed in 12 community settings (e.g. grocery stores, local eateries, clinics, mosques, churches, and community events) across three MI counties. The study was promoted through posters, fliers and additional in‐person outreach. Participants provided informed consent and were given the option of completing the survey with a pen and paper or online, with assistance if needed from trained, bilingual interviewers, and were given a $25 monetary incentive. The final sample includes 286 Arab respondents with fully completed surveys. The study was approved by the Institutional Review Board (IRB) at Elon University.

### 2.2 Measures

#### 2.2.1 Depression and Anxiety

Depression and anxiety were measured with the four‐item PHQ‐4 scale (*α* = 0.94), which has a depression subscale (*α* = 0.89) and an anxiety subscale (*α* = 0.89), each with two items (Kroenke et al. 2009). Participants were asked to indicate the frequency (“not at all” ( = 0) to “nearly every day” ( = 3)) of the following statements: “over the past 2 weeks, how often have you been bothered by any of the following problems: Little interest or pleasure in doing things; feeling down, depressed, or hopeless (depression); feeling nervous, anxious, or on edge; and not being able to stop or control worrying? (anxiety).” We treated depression and anxiety as a latent variable derived from the four observed scale indicators. This allowed us to incorporate measurement error and the covariance between these indicators in the model.

#### 2.2.2 Discrimination

Experiences of discrimination was measured with a combined 12‐item scale (*α* = 0.94) made up of nine items measuring chronic discrimination stressors (Williams et al. 1997) and three items measuring major life events (Rayman 2016). The statements on the scale for chronic discrimination were: “you are treated with less courtesy than other people are; you are treated with less respect than other people are; you receive poorer service than other people at restaurants or stores; people act as if they think you are not smart; people act as if they are afraid of you; people act as if they think you are dishonest; people act as if they're better than you are; you are called names or insulted; and you are threatened or harassed.” The items measuring major life events were: “you were denied housing; you were hassled by law enforcement (including TSA, police, etc.); and you were denied/fired from a job.” Participants selected the frequency of these events, from “less than once a year” ( = 1) to almost every day ( = 6). To account for missing data, we used the mean score across the 12 items for each participant (range 1–5.3).

#### 2.2.3 Ethnic Identity

Ethnic identity was measured with a 20‐item scale (Resnicow et al. 2021) with a Cronbach's alpha of 0.92. The scale was originally developed for African Americans and then adapted for Arab Americans (Resnicow et al. 2021). The items include, “both in my public and private thoughts, being Arab American is an important part of who I am; many things that make me happy are connected to the fact that I am Arab American; many things that are important to me are connected to my Arab American identity; I feel a strong emotional connection to the Middle East or North Africa; it is important for Arab American people to educate their children about Arab/Arab American art, history, music, and literature; I have a strong sense of belonging to the Arab American community,” (see Supplemental Material for the full list of items in the scale). Participants indicated agreement or disagreement with the statements on a 4‐point scale from strongly disagree to strongly agree, and a higher score indicated a stronger sense of ethnic identity (range 1–4). In the structural models, we evaluated ethnic identity as a moderator of the theorized relationship between discrimination and latent depression/anxiety using a continuous summed variable for the analysis test of moderation and for the multiple group analysis test of moderated moderation. We used an interaction term of ethnic identity and discrimination for the moderated moderation models. We used the total scale score rather than the subscales to limit the number of moderator effects in the model, as recommended for this type of analysis with the scale (Resnicow et al. 2021).

#### 2.2.4 Additional Measures

Sex was measured dichotomously (male/female) and is used as a proxy for gender in this study. Table 1 contains the ranges, means, standard deviations, and correlations of the key independent and dependent variables. Estimates from the Arab American Institute were used to determine the Arab American population and country‐of‐origin sub‐groups in Michigan (Arab American Institute Foundation 2021). Data were then weighted using post‐stratification ranking based on mother's and father's country of origin to be more representative of the Arab American population distribution in SE Michigan based on estimates from the Arab American Institute Foundation (2021). Weighted data were used for demographic percentages, prevalence of predictor and outcome variables.

**Table 1:** Ranges, means, standard deviations, and correlations of independent and dependent scales.

Variables	Range	Mean	SD	1	2
1. Discrimination	1–6	1.68	1.15		
2. Ethnic identity	1–4	3.00	0.99	−0.026	
3. Anxiety & depressive symptoms	0–3	0.71	0.96	0.24	−0.12

### 2.3 Statistical Analysis

**Figure 1:**
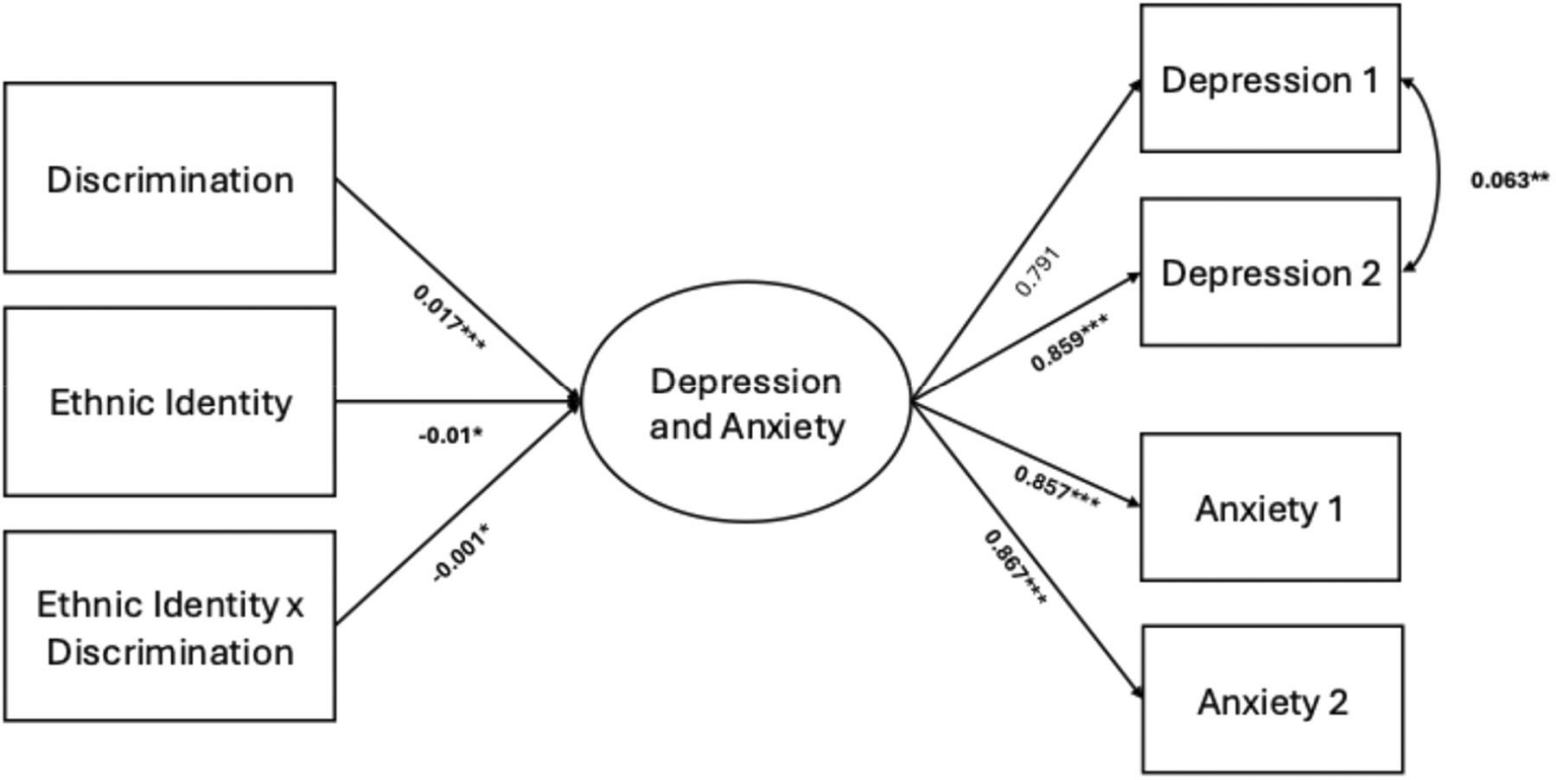
Standardized structural equation moderation model of ethnic identity.

To assess the effects of ethnic identity on the relationship between discrimination and latent depression and anxiety, we tested the statistical significance of the path coefficients with path and multiple group analysis of the moderation model (Fairchild and MacKinnon 2009). We examined sex as a further moderator in this model using multiple group analysis to explore tests of statistical significance for path coefficients (Cohen et al. 2013). Multiple group analysis tests whether sub‐groups within the data fit the same overall model, and whether the relationships in the model, including for latent variables, have the same significance and strength for the sub‐groups (Evermann 2010). In multiple group analysis, separate chi‐square values and model parameters are calculated for each group and the models are then permitted to vary by group (Jöreskog 1971; Sörbom 1974). We tested the moderation model using a continuous Ethnic Identity variable. For the second model we created an interaction term of (Ethnic Identity x Discrimination) with mean‐centered variables (Aiken et al. 1991) and used multiple group analysis with male and female subgroups. Neither model included control variables as the models became over‐identified. We tested the existence of relationships between path coefficients and then conducted Wald tests of constraint for the significant interaction terms, shown Table 4. Standardized path models for ethnic identity moderation are included in Figure 1. All analysis were conducted using the lavvan package in R statistical software version 1.3.959 (Rosseel 2012).

## 3 Results

Descriptive statistics for the sample are in Table 2. The majority (94%) of respondents were Muslim, 64% identified as female, and 35.3% were US‐born. Nearly half (48.8%) of respondents were 18–29 years old, 25.8% were 30–44 years old, 19.8% were 45–59 years old, and 5.7% were aged 60 & older. About half of the respondents were employed (46.5%), while 21.1% reported they were unemployed and 17.3% listed their occupation as student. Of the sample, 62.6% reported an annual income of less than $35,000. 41.8% of respondents reported they had earned a college or graduate degree and 25.2% had an associate degree or had completed some college education.

**Table 2:** Selection of key demographic variables.

Demographic variables	Total sample (*N *= 286)	
	Unweighted *n*	Weighted %
**Sex *N* (weighted %)**	**286**	
Female	176	(64.7)
Male	110	(35.4)
**Religion**	**281**	
Muslim	258	(93.6)
Christian	13	(4.6)
Other	6	(0.36)
None	4	(1.4)
**Nation of ancestry**	**286**	
Iraq	25	(6.0)
Lebanon or Syria	165	(40.9)
Yemen	61	(35.6)
USA	1	(0.70)
Other (Palestine, Syria, Morocco, Saudi Arabia, Egypt)	21	(12.3)
Mixed ancestry	13	(4.6)
**Immigration status**	**284**	
US‐born	108	(35.3)
Foreign‐born	176	(64.7)
**Age**	**281**	
18–29	147	(48.8)
30–45	64	(25.8)
46–64	54	(19.8)
65 and older	16	(5.7)
**Employment status**	**280**	
Employed	136	(46.5)
Homemaker	23	(8.5)
Student	52	(17.3)
Other	15	(6.7)
Unemployed	54	(21.1)
**Education level**	**279**	
High school or less	16	(13.5)
Completed high school	54	(19.5)
Associates' degree/some college	74	(25.2)
Completed college	89	(31.9)
Postgraduate education	29	(9.9)
**Income**	**273**	
$0–14,999	78	(29.0)
$15,000–34,999	89	(33.6)
$35,000–74,999	66	(24.4)
$75,000+	38	(13.1)
**Language(s) spoken at home**	**271**	
Arabic	58	(24.7)
English	190	(67.9)
Other	23	(7.4)

### 3.1 Ethnic Identity

We tested two models of ethnic identity: moderation (EI changes relationship between discrimination and latent depression and anxiety) and moderated moderation with sex (the interaction effect of EI and Discrimination differs between male and female participants). Table 3 includes the model fit indices for all models, and these were all conducted using the Full Information Maximum Likelihood (FIML) estimation.

**Table 3:** Model fit indices of the moderation and moderated moderation with multiple group analysis for ethnic identity.

Model	*χ* ^2^ (df) [ > 0.95, *p* > 0.05]	CFI [> 0.95]	TLI [> 0.95]	RMSEA [< 0.08]	SRMR [< 0.08]
***Ethnic identity***					
1. EI Moderation	9.73 (10)	1.00	1.00	0.000	0.015
2. EI x Sex moderated moderation	25.70 (20)	0.994	0.989	0.045	0.021
*Female group*	11783	—	—	—	—
*Male group*	13.92	—	—	—	—

*Note:* Boldface font indicates statistical significance (*p < 0.05, **<0.01, ***<0.001).

#### 3.1.1 Moderation

The moderation model had a total of 10 missing observations. With all five fit indices taken together, the model offers a good fit for this data. These data, along with thresholds for good model fit, can be found in Table 3. We examined the path coefficients to latent depression and anxiety for discrimination, ethnic identity, and the interaction of the two. Discrimination was positively associated with latent depression and anxiety (B = 0.017, *p* = 0.000) and ethnic identity was negatively associated (B = −0.01, *p* = 0.03). The interaction term was negatively associated, wherein the relationship between discrimination and latent depression and anxiety decreased for every unit increase in ethnic identity. Wald test results to confirm these effects are included in Table 4.

**Table 4:** Interaction regression coefficients and confidence intervals for moderation and moderated moderation for predicting depression and anxiety for ethnic identity.

Predictors	*B* a	*SE* b	ß^c^	*p* value	95% CI	Wald *χ* ^2^ test of parameter constraints
***Ethnic identity***						
Ethnic identity × discrimination	−0.001*	0.00	**−**0.151	0.022	(**−**0.002, **−**0.00)	4.97*
*Female*						
Ethnic identity × discrimination	−0.001*	0.001	**−**0.179	0.035	(**−**0.002, **−**0.00)	4.25*
*Male*						
Ethnic identity × discrimination	**−**0.001	0.001	**−**0.163	0.144	(**−**0.003, 0.000)	1.90

*Note:* Boldface font indicates statistical significance (*p < 0.05, **<0.01, ***<0.001).

^a^ *Unstandardized beta coefficients.*

^b^ *Standard errors.*

^c^ *Standardized factor loadings for EI and CEI indicators.*

#### 3.1.2 Moderated Moderation‐Sex

The moderated moderation model with sex had 20 missing observations. All five model fit indices met the criteria for good fit (*χ* ^2^ = 25.70, df = 20, *p* = 0.18, RMSEA = 0.045, SRMR = 0.021, CFI = 0.994, TLI = 0.989) and the modification indices offered no significant or meaningful improvements for this model.

In the male sub‐group, there was no joint effect of EI and discrimination on latent depression and anxiety, though both discrimination (B = 0.017, *p* < 0.001) and EI (−0.018, *p* = 0.008) were statistically significantly associated with latent depression and anxiety. In the female sub‐group, the main effect path coefficient for EI was not statistically significantly associated with latent depression and anxiety, though the discrimination was negatively associated (B = 0.016, *p* = 0.005) and the interaction term of EI x Discrimination was significant and negatively associated with latent depression and anxiety (B = −0.001, *p* = 0.035). A Wald test confirmed that the interaction effect was significantly different from zero among female participants (*χ* ^2^ = 4.25, *p* = 0.039, df = 1). A stronger sense of EI was protective against poor mental health outcomes from discrimination among female participants.

We further tested the interaction effects among males and females in the group using simple slopes analysis and Johnson‐Neyman test with a continuous EI variable. The tests of simple slopes among female participants showed a statistically significant and positive relationship with Discrimination and lower values of EI at the mean and 1 SD below the mean (‐1 SD: t = 3.96, *p* = 0.00; the mean: t = 2.62, *p* = 0.01). The higher level of EI at 1 SD above the mean did not have a statistically significant relationship with Discrimination ( + 1 SD: t = 0.27, *p* = 0.79). Among male participants, the simple slopes analysis showed a statistically significant and positive relationship between Discrimination and lower values of EI (‐1SD: t = 1.87, *p* = 0.06; the mean: t = 2.13, *p* = 0.04). A higher level of EI did not have a statistically significant relationship with Discrimination ( + 1 SD: t = 0.66, *p* = 0.51). The Johnson‐Neyman interval shows the effect of Discrimination is only significant when EI is lower than 64.06 among female participants, while the effect of Discrimination among male participants is only significant in the interval of 51.09–62.59. See the supplementary material, Figure 2, for the Johnson‐Neyman plots.

## 4 Discussion

Our objective was to assess the possible buffering of ethnic identity on the relationship between discrimination and depression and anxiety and second‐level moderation of sex in a sample of Arab American adults in SE Michigan. The hypothesis of ethnic identity changing the relationship between discrimination and poor health outcomes was confirmed. There was a significant association between the interaction of discrimination and EI and depression and anxiety. This finding provides evidence for the protective nature of ethnic identity among Arab American adults in the ethnic community. It aligns with studies that have found a strong sense of ethnic identity is protective against poor mental health outcomes associated with discrimination among other ethnic minority groups, including African Americans, Latinos, and Asian Americans (Espinosa et al. 2018; Tynes et al. 2012).

The protective nature of ethnic identity may function on both a personal and a community level for Arab Americans in SE Michigan. At the individual level, the effect may be due to feelings of belonging, purpose, meaning, and self‐esteem among Arab Americans in this community (Espinosa et al. 2018; Sheldon et al. 2015), bolstered by the effects of living in an ethnic enclave, which can help protect against negative effects of discrimination. Individuals with a strong sense of ethnic identity have a positive construction of this identity and therefore may not internalize experiences of discrimination (Sellers et al. 2006; Tynes et al. 2012). A strong sense of ethnic identity may also give individuals hope and a sense of purpose (Kumar et al. 2014). This strong sense of ethnic identity may be especially important for those Arab Americans who don't have access to acculturation to mainstream US culture due to discrimination and perceived “otherness.” While there was previous understanding around ethnic identity among Arab American adolescents (see Seff et al. 2021; Kumar et al. 2014, 2015), this finding around the importance and protective effects of ethnic identity for mental health among adults in the ethnic enclave community adds to our understanding of discrimination and mental health for Arab Americans more generally along with the importance of the ethnic enclave as a supportive space for that identity maintenance.

Though the EI scale was measured at the individual level, it encompasses multidimensional elements of ethnic identity, including items related to community and cultural involvement (e.g. “I have a strong sense of belonging to the Arab American community,” “It is important to be involved in the Arab American community,” “It is important for Arab American people to educate their children about Arab/Arab American art, history, music, and literature”) (Resnicow et al. 2021). Residence in an area with other Arab Americans may help residents in Dearborn develop and maintain a positive view of their ethnic identity through community involvement. This residence can also provide a sense of connection and belonging to home culture without having to return to the homeland (Suarez‐Orozco 2004).

A sense of social cohesion, or feelings of connectedness and belonging, which is often present in ethnic enclave communities can play an important role in the health and well‐being of their members (Bjornstrom et al. 2013; Jang et al. 2015). In previous studies, researchers have found that a strong sense of community and participation in community life are directly related to positive ethnic identity and act as protective factors against poor mental health (Garcia‐Reid et al. 2013; Lardier 2018). At the community level, ethnic identity can be embodied by social support and resources and a shared worldview (Branscombe et al. 1999), elements of which can exist in ethnic enclave communities. Researchers have shown that the role of ethnic enclaves has moved beyond the traditional understanding as primarily economic resources to being spaces of cultural and community history and life, as well as spaces of activism and resistance where residents can resist the inequality that may confront them in dominant cultural and social spaces (Liu and Geron 2008). In research on a Guyanese ethnic enclave, Bacchus found residents used elements of their community for cultural attachment and shaping their identity (Bacchus 2020). Arab Americans in SE Michigan reside in an area with one of the highest concentrations of Arab Americans in the US (Arab American Institute Foundation 2018). The opportunity for community and cultural involvement is high in the area, and as a result, Arab American adults in this community may be especially able to develop and maintain a strong ethnic identity and access social support and other protective aspects of ethnic identity at the community level, with possible benefits indicated in our results including coping with discrimination and resulting poor mental health outcomes.

Within the sub‐group analyses, sex played a complex role. Ethnic identity was protective against poor mental health associated with discrimination for female participants, though not male participants. As the majority of the sample was Muslim, female participants may be both more identifiable as (and conflated) as Muslim and Arab (Gulamhussein and Eaton 2015) and may also be more marginalized from and discriminated against in mainstream society (Cainkar 2009). For some, this may have resulted in an embrace of Arab American identity as a protective factor against this marginalization (Awad 2010). The protective nature of ethnic identity on depression and anxiety associated with discrimination for women in the sample may also stem from their role as holders and transmitters of Arab culture and religious traditions (Samari 2016). Many women are expected by their families and communities to uphold Arab traditions and pass them to the next generation and are encouraged to learn about and embrace their culture and Arab identity (Read and Oselin 2008). This may further instill a strong sense of and positive association with their Arab ethnic identity.

The coping mechanisms women are using to deal with experiences of discrimination may differ than those used by men in the same community. Women may be more likely than men to utilize social support to cope with experiences of discrimination (Assari and Lankarani 2017). For women in this enclave community, social support may also be tied to ethnic identity and/or connections with cultural resources, as it was among MENA college students in a study in California (Modir and Kia‐Keating 2018). Women may also be utilizing more communal coping to deal with discrimination, leading to a positive association with their ethnic identity, similar to what Latina women facing discrimination reported in a recent study (Sanchez et al. 2018). These findings around ethnic identity differences by sex do align with a study that found higher levels of Arab cultural practices among female respondents compared to male respondents (Amer and Hovey 2007) and another which found higher private regard of Arab American identity among female respondents compared to male respondents (Ahmed et al. 2022). The buffering role of ethnic identity among women may also relate to the expectation among many in the Arab American community for women to maintain Arab identity and help children develop their Arab identity, especially when that identity is seen to stand in contrast to larger societal and social environmental factors (Read and Oselin 2008).

There may be other characteristics besides gender alone that account for these differences between women and men in the sample. Acculturation patterns have been found to differ between Arab American men and women, with men reporting higher levels of acculturation to dominant US culture compared to women (Sadek and Awad 2024). Men may feel less pressure to maintain traditional Arab culture, as the community expectation is for them to fulfill the American dream and be financially successful (Ajrouch 2000) and may be experiencing less detrimental discrimination as a result. Many women in the Arab American community, particularly those who are immigrants or first‐generation, elect to not work outside the home and instead focus on domestic tasks (Bulut and Carlson 2020), which could help further explain the buffering role of ethnic identity between discrimination and poor mental health outcomes for women. More research around gender, including roles and expectations, in connection to discrimination, ethnic identity, and mental health for adult Arab American men is needed to better understand these relationships.

This analysis has several limitations. The data come from a cross‐sectional community convenience sample which limits generalizability, particularly outside of SE Michigan. For example, our sample skewed towards younger participants, those with lower incomes, and a higher percentage of those with Lebanese and Yemini ancestry compared to national Arab American populations (Arab American Institute Foundation 2018; Hekman et al. 2015). A second limitation comes from the moderate sample size. The number of participants in the sample limited the types of analysis and models that could be run using structural equation modeling. The power for parameter estimation of the overall moderation model was 0.98 for the effect between Discrimination and latent Depression and Anxiety, 0.73 for the effect of interaction term of Ethnic Identity and Discrimination on latent Depression and Anxiety and 0.75 for the effect of Ethnic Identity alone on latent Depression and Anxiety (Wang and Rhemtulla 2021). This may have influenced the effect sizes in the models and a larger sample size may have improved the power. Additionally, we were not able to add additional control measures into the models as they became under‐identified and would offer no additional insight than traditional regression models. As a result, the models were not able to account for all the variation in the data and some portion of the effects may also be due to differences in religion, income, national origin, or other variables. There were also measurement limitations. The EI scale is newly developed and has only been used within this sample, though the scale did have high internal consistency and removal of items did not improve alpha scores. Further testing and validation of this measurement in other MENA populations would be beneficial. While we did not disaggregate the data by the measurement subscales, doing so may have resulted in a different pattern of associations (see Table 5 in the Supplementary Material). We also measured sex in our survey, though the sex with which participants identified may not have aligned with their gender identity. We may have missed out on important data around sex and gender identity and the relationship that may have with discrimination, ethnic identity, and mental health, however, this is line with other studies among the Arab American population (i.e. Samari 2016; Assari and Lankarani 2017; Ikizler and Szymanski 2018), in which sex is treated as a socio‐demographic variable. Additionally, we did not have a study variable to look at language use which may be related to discrimination, acculturation, and ethnic identity. Finally, based on our cognitive pre‐testing of the survey where respondents were unable to distinguish between ‘never’ and ‘less than once per year’ in the Everyday Discrimination Scale, we did not include a ‘never’ response option and would have perhaps had different results if we had used the traditional scale.

Even with these limitations, this study and offer important insight into ethnic identity as a protective element for mental health among Arab American adults. This is the first study examine the effects of ethnic identity on discrimination and mental health among Arab American adults living in the ethnic enclave community in SE Michigan. We were able to examine depression and anxiety as a latent variable, allowing for a more thorough understanding of how ethnic identity influences mental health outcomes. Future research should explore if these effects differ for Arab Americans based on national origin, religion, education, income, or other demographic factors. Though this study was specific to the Arab American community, the effects of the protective role of ethnic identity should be further explore in the broader MENA community, as there may be similar effects for the community overall.

Many Arab Americans experience discrimination that negatively affect their mental health. The community faces rates of poor mental health similar to other ethnic minority communities. However, positive aspects of the Arab American identity that accompany it can counter these stressors and help promote positive mental health. Activities which build and reaffirm Arab ethnic identity should be emphasized in interventions to improve mental health in the community and by providers as a resource for patients. While women who assign importance to their Arab identity are likely able to access protective elements in the community, for men in the community, the protective relationship of ethnic identity for negative mental health effects associated with discrimination may not function in the same way as it does for women. Providers and those conducting mental health intervention work should understand that gender and ethnic identity as a coping mechanism against discrimination may be linked. Approaches to improve mental health outcomes for men need to take this into account and find creative ways to facilitate the access to and use of community‐based coping resources among Arab American men. Implementing approaches that utilize community‐based resources and social support, including through group‐based interventions and community wide changes as preventative measures to improve mental health, may increase accessibility and acceptability for mental healthcare in the community (The Prevention Institute 2014; Walter et al. 2017).

Efforts to address mental health disparities, decrease stigma, and increase treatment for mental health issues for Arab Americans should focus on incorporating elements of Arab ethnic identity and community to promote positive mental health. Doing so will help individuals in the community to live healthier lives and this diverse and growing community to further thrive and flourish. With this type of approach, community members will have the support needed to continue to make changes that benefit the Arab American community in SE Michigan and throughout the country.

## Ethics Statement

The study was approved by the Institutional Review Board (IRB) at the University of Michigan.

## Conflicts of Interest

The authors declare no conflicts of interest.

## Supporting information


Supplementary Material

## Data Availability

The data that support the findings of this study are available from the corresponding author upon reasonable request. Data sharing via repositories supported, in accordance with University of Michigan's institutional data sharing policies.
