# Supplementary material for: The Effects of Ethnic Identity on Discrimination and Depression and Anxiety in a Sample of Arab American Adults
Source: J Community Psychol. 2025 Dec 2;54(1):e70068. doi: 10.1002/jcop.70068 (PMC12671900; doi:10.1002/jcop.70068)
Supplement: Supplementary file 1 — Supplementary Material [file JCOP-54-0-s001.docx]

**Supplementary Material**

**Full Ethnic Identity Measure**

| **1.** When I listen to the radio, I usually listen to Arab American radio shows, e.g., CINA |
| --- |
| **2.** When I watch television, I usually watch Arabic television shows, such as ART and MBC |
| **3.** When I look for news, I read mostly Arabic news such as The Arab American News and the Dearborn Facebook page |
| **4.** In my private thoughts, I think of myself more as Arab American than American |
| **5.** Both in my public and private thoughts, being Arab American is an important part of who I am |
| **6.** Many things that make me happy are connected to the fact that I am Arab American |
| **7.** Many things that are important to me are connected to my Arab American identity |
| **8.** I feel a strong emotional connection to the Middle East or North Africa |
| **9.** Arab Americans should give their children Arabic names |
| **10.** It is important for us to eat Middle Eastern/North African food at home |
| **11.** It is important for Arab Americans to get back to their Middle Eastern/North African roots |
| **12.** A thorough knowledge of Arab and Arab American history is very important for our community today |
| **13.** It is important for Arab American people to educate their children about Arab/Arab American art, history, music, and literature |
| **14.** I have a strong sense of belonging to the Arab American community |
| **15.** It is important to be involved in the Arab American community |
| **16.** I feel strongly about international human rights issues in places such as Africa |
| **17.** Most of my friends are Arab American |
| **18.** I care deeply about the needs of other groups such as Native Americans, African Americans, Latinos, and Asian Americans |
| **19.** I respect the cultural traditions of many groups-for example Native Americans, African Americans, Latinos, And Asian Americans |
| **20.** I keep up with political activities in the Middle East and North Africa |

**Figure 2. Graph of Johnson-Neyman analysis for male and female participants**

**
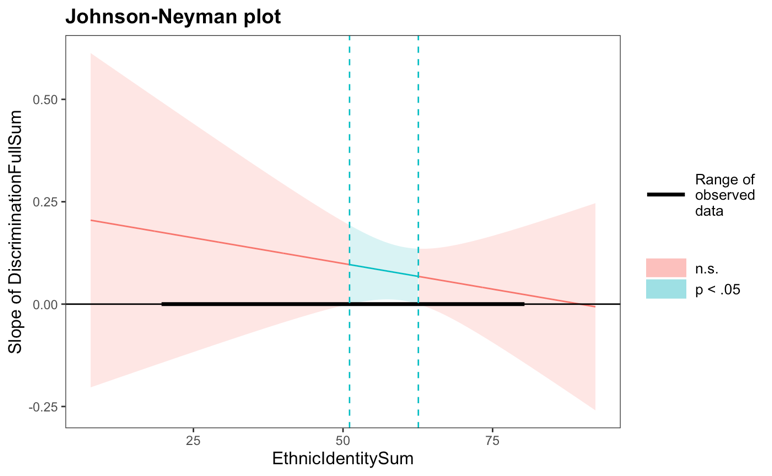
**

Male participants

**
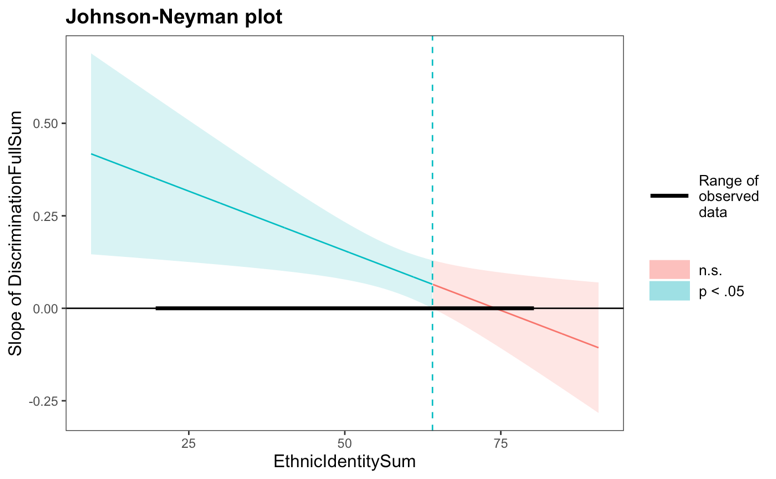
**

Female participants

**Table 5. Identity Measure Subscale Correlations**

|  | Subscale 1-Cultural Affiliation | Subscale 2-Media | Subscale 3-Multicultural |  |  |
| --- | --- | --- | --- | --- | --- |
| Cultural Affiliation |  |  |  |  |  |
| Media | 0.36 |  |  |  |  |
| Multicultural | 0.58 | 0.13 |  |  |  |
|  |  | | |  |  |
| Discrimination | -0.08 | 0.05 | -0.06 |  |  |
| Depression and Anxiety | -0.07 | -0.06 | -0.14 |  |  |

*note: the correlations were calculated using the summed scores of the subscales
